# Supplementary material for: Hot spot prediction in protein-protein interactions by an ensemble system
Source: BMC Syst Biol. 2018 Dec 31;12(Suppl 9):132. doi: 10.1186/s12918-018-0665-8 (PMC6311905; doi:10.1186/s12918-018-0665-8)
Supplement: Supplementary file 2 — All relevant MATLAB code. A simple Matlab implement of our predictor. (ZIP 19,742 kb) [file 12918_2018_665_MOESM2_ESM.zip › Additional file 2/Read ASA/ASA website/dbMPIKT/5K39B.html]

 NetSurfP - Protein Surface Accessibility and Secondary Structure Predictions

  

|  |  |  |
| --- | --- | --- |
|  |  | NetSurfP - Protein Surface Accessibility and Secondary Structure Predictions  Technical University of Denmark |

  


---

```
# For publication of results, please cite:
# A generic method for assignment of reliability scores applied to solvent accessibility predictions.
# Bent Petersen, Thomas Nordahl Petersen, Pernille Andersen, Morten Nielsen and Claus Lundegaard
# BMC Structural Biology 2009, 9:51 doi:10.1186/1472-6807-9-51
#
# Column 1: Class assignment - B for buried or E for Exposed - Threshold: 25% exposure, but not based on RSA
# Column 2: Amino acid
# Column 3: Sequence name
# Column 4: Amino acid number
# Column 5: Relative Surface Accessibility - RSA
# Column 6: Absolute Surface Accessibility
# Column 7: Z-fit score for RSA prediction
# Column 8: Probability for Alpha-Helix
# Column 9: Probability for Beta-strand
# Column 10: Probability for Coil
E M  5K39B                  1    0.822 164.482  -0.803   0.003   0.003   0.994
E N  5K39B                  2    0.730 106.901  -1.279   0.018   0.047   0.935
E N  5K39B                  3    0.522  76.450  -0.791   0.018   0.047   0.935
E D  5K39B                  4    0.639  92.037  -1.115   0.053   0.043   0.903
E S  5K39B                  5    0.338  39.614  -0.695   0.052   0.084   0.864
E T  5K39B                  6    0.390  54.162  -0.803   0.056   0.142   0.802
E D  5K39B                  7    0.671  96.648  -0.361   0.056   0.142   0.802
E K  5K39B                  8    0.487 100.217   0.146   0.019   0.141   0.840
B T  5K39B                  9    0.178  24.689   0.292   0.021   0.451   0.528
E T  5K39B                 10    0.305  42.303   0.097   0.003   0.718   0.279
B V  5K39B                 11    0.058   8.838  -0.407   0.002   0.816   0.182
B S  5K39B                 12    0.096  11.216  -0.564   0.003   0.718   0.279
B G  5K39B                 13    0.050   3.927  -1.367   0.003   0.718   0.279
B Y  5K39B                 14    0.149  31.948  -0.358   0.002   0.816   0.182
B I  5K39B                 15    0.072  13.394  -0.347   0.003   0.718   0.279
B S  5K39B                 16    0.195  22.889  -0.094   0.005   0.336   0.660
B V  5K39B                 17    0.254  39.101  -1.054   0.018   0.088   0.893
E D  5K39B                 18    0.435  62.640  -0.782   0.018   0.047   0.935
B F  5K39B                 19    0.117  23.542  -0.013   0.018   0.088   0.893
E D  5K39B                 20    0.537  77.396  -0.826   0.004   0.138   0.858
B Y  5K39B                 21    0.267  57.015   0.329   0.018   0.088   0.893
E P  5K39B                 22    0.451  64.039  -0.611   0.018   0.019   0.964
E P  5K39B                 23    0.560  79.421  -0.867   0.115   0.016   0.868
E E  5K39B                 24    0.621 108.524  -0.613   0.268   0.043   0.689
E S  5K39B                 25    0.291  34.105  -0.154   0.354   0.048   0.598
E E  5K39B                 26    0.283  49.388  -0.152   0.455   0.046   0.498
E S  5K39B                 27    0.447  52.424  -0.528   0.354   0.048   0.598
E K  5K39B                 28    0.285  58.583   0.078   0.428   0.171   0.402
B I  5K39B                 29    0.102  18.815  -0.020   0.352   0.332   0.316
E K  5K39B                 30    0.442  90.961   0.613   0.231   0.330   0.439
E S  5K39B                 31    0.337  39.461  -1.110   0.125   0.227   0.648
E G  5K39B                 32    0.307  24.145  -1.771   0.020   0.205   0.775
B F  5K39B                 33    0.083  16.738  -0.011   0.022   0.359   0.619
E N  5K39B                 34    0.368  53.817   0.792   0.003   0.718   0.279
B V  5K39B                 35    0.066  10.175   0.492   0.011   0.918   0.071
E K  5K39B                 36    0.327  67.182   0.563   0.006   0.962   0.032
B V  5K39B                 37    0.114  17.537   0.139   0.001   0.959   0.040
B A  5K39B                 38    0.262  28.894   0.238   0.001   0.900   0.099
B G  5K39B                 39    0.223  17.534  -1.177   0.003   0.718   0.279
B T  5K39B                 40    0.254  35.230  -0.152   0.004   0.616   0.381
E E  5K39B                 41    0.477  83.314  -0.662   0.021   0.756   0.223
B L  5K39B                 42    0.268  49.107   0.182   0.023   0.655   0.322
B S  5K39B                 43    0.260  30.495  -0.234   0.023   0.655   0.322
E T  5K39B                 44    0.369  51.139  -0.535   0.023   0.655   0.322
B K  5K39B                 45    0.286  58.748   0.088   0.021   0.756   0.223
E T  5K39B                 46    0.324  44.953  -0.455   0.023   0.655   0.322
E D  5K39B                 47    0.391  56.372  -0.655   0.021   0.279   0.699
E E  5K39B                 48    0.617 107.755  -1.765   0.018   0.088   0.893
E K  5K39B                 49    0.566 116.488  -0.001   0.018   0.088   0.893
B G  5K39B                 50    0.134  10.562  -1.029   0.005   0.262   0.733
B Y  5K39B                 51    0.244  52.228   0.255   0.003   0.718   0.279
B F  5K39B                 52    0.091  18.264  -0.048   0.011   0.918   0.071
E E  5K39B                 53    0.268  46.785   0.464   0.011   0.918   0.071
B I  5K39B                 54    0.064  11.859  -0.202   0.018   0.846   0.136
E S  5K39B                 55    0.409  47.923   0.338   0.004   0.616   0.381
B G  5K39B                 56    0.299  23.523  -1.597   0.004   0.420   0.576
B I  5K39B                 57    0.133  24.550   0.775   0.005   0.336   0.660
B P  5K39B                 58    0.268  38.015  -0.715   0.019   0.141   0.840
E G  5K39B                 59    0.522  41.089  -1.750   0.018   0.047   0.935
E D  5K39B                 60    0.499  71.964  -1.697   0.058   0.017   0.925
B M  5K39B                 61    0.189  37.739  -1.027   0.052   0.084   0.864
E R  5K39B                 62    0.348  79.784  -0.547   0.022   0.359   0.619
E E  5K39B                 63    0.386  67.364  -0.548   0.022   0.552   0.426
B F  5K39B                 64    0.122  24.485   0.560   0.021   0.756   0.223
B T  5K39B                 65    0.117  16.228  -0.033   0.018   0.846   0.136
B L  5K39B                 66    0.076  13.879   0.191   0.011   0.918   0.071
E E  5K39B                 67    0.363  63.451   1.094   0.011   0.918   0.071
B I  5K39B                 68    0.104  19.166   0.001   0.018   0.846   0.136
B S  5K39B                 69    0.309  36.203  -1.009   0.022   0.552   0.426
E K  5K39B                 70    0.392  80.614   0.129   0.066   0.296   0.638
E R  5K39B                 71    0.416  95.150  -0.429   0.125   0.227   0.648
E N  5K39B                 72    0.355  51.899  -0.279   0.125   0.227   0.648
B Y  5K39B                 73    0.176  37.633  -0.139   0.135   0.317   0.548
B L  5K39B                 74    0.081  14.868  -0.396   0.148   0.418   0.435
E K  5K39B                 75    0.479  98.592   0.769   0.074   0.484   0.442
B R  5K39B                 76    0.212  48.617   0.191   0.087   0.683   0.230
E N  5K39B                 77    0.308  45.135   0.008   0.018   0.846   0.136
B V  5K39B                 78    0.137  21.042   0.225   0.011   0.918   0.071
B T  5K39B                 79    0.171  23.718  -0.360   0.011   0.918   0.071
B V  5K39B                 80    0.070  10.744  -0.725   0.011   0.918   0.071
B N  5K39B                 81    0.185  27.143  -0.119   0.011   0.918   0.071
B G  5K39B                 82    0.107   8.445  -0.307   0.011   0.918   0.071
B T  5K39B                 83    0.190  26.422  -0.777   0.018   0.846   0.136
B G  5K39B                 84    0.183  14.441  -1.204   0.021   0.756   0.223
E K  5K39B                 85    0.433  89.109   0.228   0.023   0.655   0.322
B L  5K39B                 86    0.233  42.699  -0.245   0.021   0.756   0.223
B V  5K39B                 87    0.313  48.139  -1.137   0.018   0.846   0.136
B V  5K39B                 88    0.160  24.653  -0.200   0.021   0.756   0.223
B S  5K39B                 89    0.303  35.523  -0.450   0.023   0.655   0.322
B T  5K39B                 90    0.246  34.092  -0.357   0.022   0.552   0.426
E E  5K39B                 91    0.415  72.431  -1.048   0.066   0.296   0.638
E D  5K39B                 92    0.557  80.206  -1.266   0.056   0.142   0.802
E N  5K39B                 93    0.369  54.022  -0.598   0.052   0.084   0.864
B P  5K39B                 94    0.229  32.467  -1.015   0.064   0.216   0.721
B L  5K39B                 95    0.132  24.096   0.319   0.022   0.552   0.426
B I  5K39B                 96    0.131  24.327   0.638   0.021   0.756   0.223
B L  5K39B                 97    0.060  10.986   0.533   0.021   0.756   0.223
B W  5K39B                 98    0.091  21.861  -0.406   0.087   0.683   0.230
B A  5K39B                 99    0.046   5.124  -1.148   0.079   0.592   0.329
B G  5K39B                100    0.093   7.343  -1.521   0.069   0.386   0.545
B D  5K39B                101    0.237  34.123  -0.684   0.069   0.386   0.545
B V  5K39B                102    0.071  10.913   0.044   0.022   0.552   0.426
E E  5K39B                103    0.293  51.117   0.103   0.022   0.552   0.426
E R  5K39B                104    0.333  76.234  -0.277   0.069   0.386   0.545
E K  5K39B                105    0.536 110.255  -0.267   0.064   0.216   0.721
E G  5K39B                106    0.373  29.394  -1.099   0.118   0.150   0.732
B V  5K39B                107    0.260  39.947  -0.853   0.118   0.150   0.732
E Q  5K39B                108    0.513  91.675  -0.809   0.113   0.087   0.800
E D  5K39B                109    0.491  70.768  -1.079   0.053   0.043   0.903
E N  5K39B                110    0.309  45.208  -1.303   0.113   0.087   0.800
B A  5K39B                111    0.092  10.182  -0.596   0.199   0.152   0.649
B I  5K39B                112    0.072  13.394   0.003   0.199   0.152   0.649
B N  5K39B                113    0.207  30.363   0.002   0.278   0.093   0.628
B M  5K39B                114    0.106  21.231   0.818   0.802   0.014   0.185
B V  5K39B                115    0.187  28.742  -0.359   0.879   0.010   0.111
B D  5K39B                116    0.148  21.284   0.382   0.938   0.007   0.055
B V  5K39B                117    0.025   3.827   0.835   0.975   0.003   0.022
B M  5K39B                118    0.086  17.289   0.520   0.975   0.003   0.022
B E  5K39B                119    0.176  30.765   0.612   0.975   0.003   0.022
B I  5K39B                120    0.025   4.588   0.751   0.975   0.003   0.022
B S  5K39B                121    0.039   4.583   0.060   0.970   0.001   0.030
E K  5K39B                122    0.430  88.472   0.965   0.923   0.002   0.076
B V  5K39B                123    0.111  17.107  -0.603   0.879   0.010   0.111
B F  5K39B                124    0.069  13.808   0.492   0.717   0.014   0.269
E G  5K39B                125    0.384  30.205  -0.499   0.321   0.003   0.675
E T  5K39B                126    0.426  59.156  -0.278   0.181   0.016   0.803
E R  5K39B                127    0.396  90.730  -1.792   0.115   0.016   0.868
E A  5K39B                128    0.533  58.737  -1.185   0.058   0.017   0.925
E G  5K39B                129    0.517  40.680  -1.555   0.018   0.019   0.964
E D  5K39B                130    0.300  43.244  -0.396   0.018   0.019   0.964
E E  5K39B                131    0.674 117.678  -0.658   0.181   0.016   0.803
E E  5K39B                132    0.587 102.619   0.167   0.184   0.043   0.773
B Y  5K39B                133    0.126  26.905   0.419   0.278   0.093   0.628
E V  5K39B                134    0.369  56.731  -0.154   0.321   0.252   0.427
E A  5K39B                135    0.547  60.323  -0.101   0.321   0.252   0.427
E E  5K39B                136    0.466  81.375  -0.961   0.321   0.252   0.427
B L  5K39B                137    0.193  35.302   0.057   0.216   0.235   0.548
E D  5K39B                138    0.340  48.951  -0.770   0.199   0.152   0.649
B L  5K39B                139    0.141  25.872   0.237   0.113   0.087   0.800
E N  5K39B                140    0.335  49.088  -0.478   0.053   0.043   0.903
E M  5K39B                141    0.402  80.340  -1.146   0.053   0.043   0.903
E D  5K39B                142    0.442  63.735  -1.297   0.053   0.043   0.903
B G  5K39B                143    0.129  10.176  -1.959   0.056   0.142   0.802
B A  5K39B                144    0.082   9.069  -1.107   0.066   0.296   0.638
B I  5K39B                145    0.053   9.787   0.517   0.066   0.296   0.638
B N  5K39B                146    0.149  21.843   0.671   0.199   0.152   0.649
B L  5K39B                147    0.050   9.063   0.480   0.751   0.050   0.199
B F  5K39B                148    0.179  35.925  -0.274   0.802   0.014   0.185
B D  5K39B                149    0.077  11.038  -0.989   0.879   0.010   0.111
B I  5K39B                150    0.026   4.847   0.676   0.938   0.007   0.055
B A  5K39B                151    0.060   6.568   0.058   0.950   0.028   0.022
B I  5K39B                152    0.053   9.731   0.838   0.950   0.028   0.022
B V  5K39B                153    0.024   3.704   0.709   0.938   0.007   0.055
B I  5K39B                154    0.073  13.486   0.085   0.938   0.007   0.055
E R  5K39B                155    0.418  95.814   0.662   0.879   0.010   0.111
B H  5K39B                156    0.240  43.692  -0.087   0.802   0.014   0.185
B F  5K39B                157    0.099  19.789  -0.131   0.522   0.016   0.462
E N  5K39B                158    0.517  75.630  -0.652   0.246   0.004   0.750
E A  5K39B                159    0.524  57.712   0.002   0.109   0.005   0.886
E L  5K39B                160    0.355  64.946  -0.578   0.115   0.016   0.868
E P  5K39B                161    0.502  71.248  -0.742   0.058   0.017   0.925
E S  5K39B                162    0.569  66.628  -1.567   0.058   0.017   0.925
E R  5K39B                163    0.633 144.934  -0.493   0.058   0.017   0.925
E Y  5K39B                164    0.650 138.820  -1.714   0.003   0.003   0.994

Explain the output.  Go back.
```

---
